# A qualitative, theory-based exploration of facilitators and barriers for implementation of pharmacist prescribing in chronic kidney disease

**DOI:** 10.1007/s11096-024-01794-y

**Published:** 2024-09-04

**Authors:** Fatma Al Raiisi, Scott Cunningham, Derek Stewart

**Affiliations:** 1 Oman College of Health Sciences – Pharmacy programme, Muscat, Oman; 2 Pharmacy and Life Sciences, Robert Gordon University, Aberdeen, Scotland; 3 College of Pharmacy, Qatar University, Doha, Qatar

**Keywords:** Chronic kidney disease, Clinical pharmacy, Independent prescribing, Non-medical, Pharmacist

## Abstract

**Background:**

While there is an accumulation of evidence that pharmacist prescribing is safe and effective, there is a lack of research on processes of implementation into practice, particularly for patients with complex clinical conditions such as chronic kidney disease (CKD).

**Aim:**

The aim was to explore the facilitators and barriers to the implementation of pharmacist prescribing for patients with CKD in the United Kingdom (UK).

**Method:**

Semi-structured interviews were conducted with UK Renal Pharmacy Group members who were independent prescribers. The Consolidated Framework for Implementation Research (CFIR) underpinned the interview schedule. Interviews were recorded, transcribed, and independently coded by two researchers. A thematic approach was used for analysis, with data generation continuing until saturation of themes. Ethical approval was granted.

**Results:**

Data saturation was achieved following 14 interviews. Most interviewees were female (n = 11), all had secondary care as their main practice setting, and were highly experienced prescribers with 8 having 11 or more years of prescribing practice. Interviewees were positive regarding the development of their prescribing practice. Facilitators and barriers emerged across all 5 of the CFIR domains. Key facilitators were aspects of inner setting (e.g., organisational support and communication) while key barriers were also related to inner setting, specifically the need for adequate structural and financial resources.

**Conclusion:**

This theory-based study has illuminated the facilitators and barriers for the implementation of pharmacist prescribing in CKD. There is a need to consider the resources required for implementation of prescribing practice at an early stage of planning and development.

**Supplementary Information:**

The online version contains supplementary material available at 10.1007/s11096-024-01794-y.

## Impact statements

- Grounding the research in implementation theory allowed for a comprehensive portrayal of implementation facilitators and barriers which may increase the transferability to other settings and countries.
- There is an opportunity to leverage the facilitators to further enhance implementation of pharmacist prescribing in CKD and possibly other chronic conditions.
- Barriers should be identified early in implementation planning, allowing for strategic planning to mitigate these and potentially enhance patient care and health outcomes.

## Introduction

Clinical pharmacy services significantly contribute to multidisciplinary patient management, with evidence of positive patient outcomes [1]. As stated by the European Society of Clinical Pharmacy, these services aim to ‘optimise the utilisation of medicines through practice and research in order to achieve person-centered and public health goals’ [2]. Chronic kidney disease (CKD) is associated with increased morbidity and mortality, and economic burden hence clinical pharmacy input is warranted to ensure safe and effective use of medication [3, 4]. Furthermore, most CKD patients have co-morbid conditions requiring intervention with multiple medications which potentially contribute to further risk of renal insult [5].

There are a number of models of clinical pharmacy provided to patients with CKD [6]. While systematic reviews have provided evidence of the positive impact of clinical pharmacy services on CKD outcomes, there is an acknowledged lack of theory driven studies providing in-depth understanding of service model implementation [3, 7]. Such implementation studies would allow definition and description of the key facilitators and barriers to implementation which would enable others to replicate within their practice settings [8].

Models of pharmacist prescribing have been implemented in United Kingdom (UK), Canada, United States and New Zealand [9, 10]. Pharmacist prescribing is perhaps most advanced in the UK with supplementary prescribing introduced into legislation in 2003 followed by independent prescribing in 2006 [10–12]. Until recently all pharmacist prescribers had to complete a healthcare regulator accredited postgraduate qualification [11]. Current UK undergraduate students in the initial years of their degree will have independent prescribing status on graduation [13, 14].

Systematic reviews have provided evidence of pharmacist prescribing in terms of clinical effectiveness and decision making [15–18]. These reviews have also noted that less research has been conducted on pharmacist prescribing in secondary care. The limited evidence highlights that pharmacists are prescribing in settings of inpatient wards and outpatient clinics setting for a range of medical conditions encompassing cardiology, respiratory, oncology, endocrine and renal conditions [19–21]. There is potential for expansion of secondary care pharmacist prescribing services given the UK government policies which prioritise the development of the prescribing role [22–25]. Prescribing for patients with CKD is challenging due to the complex nature of CKD and compromised health status. A recent UK survey of the implementation of pharmacist prescribing in CKD highlighted that most respondents provided general pharmaceutical care to dialysis and transplant patients, were confident in their abilities and that many were independent prescribers [26]. There is need for qualitative research in this population of pharmacist prescribers to enable in-depth understanding [27]. In particular, there is a recognised lack of qualitative research on implementation and limited use of theoretical frameworks [28]. The Consolidated Framework for Implementation Research (CFIR) is a meta-framework with five domains, identifying contextual influences that explain the heterogeneity of implementation success across settings [29]. The framework was recently updated, with minor changes in the constructs [30]. CFIR can be used to identify potential implementation barriers and facilitators which can help guide the selection of strategies to address these influential factors [29].

### Aim

The aim of this study was to explore the facilitators and barriers to the implementation of pharmacist prescribing for patients with CKD in the UK.

### Ethics approval

Approval was obtained from Robert Gordon University School of Pharmacy & Life Sciences Research Ethics Committee (Reference S172, 16/05/2018). Signed informed consent was obtained from each participant.

## Method

### Study design

This study comprised one-to-one interviews to facilitate in-depth rich data capture. Telephone interviews were selected over face-to-face interviews due to logistical issues imposed by the geographical distribution of the pharmacists.

### Setting

The study was conducted across the UK, involving members of the UK Renal Pharmacy Group (UKRPG), a non-profit organisation that aims to promote excellence in the provision of pharmaceutical services to renal patients and associated healthcare professionals [31].

### Sampling frame

UKPRG member respondents who self-identified as pharmacist prescribers during a previous survey [26] and had expressed interest in participation in further research constituted the sampling frame (n = 29).

### Recruitment

**Fig. 1:**
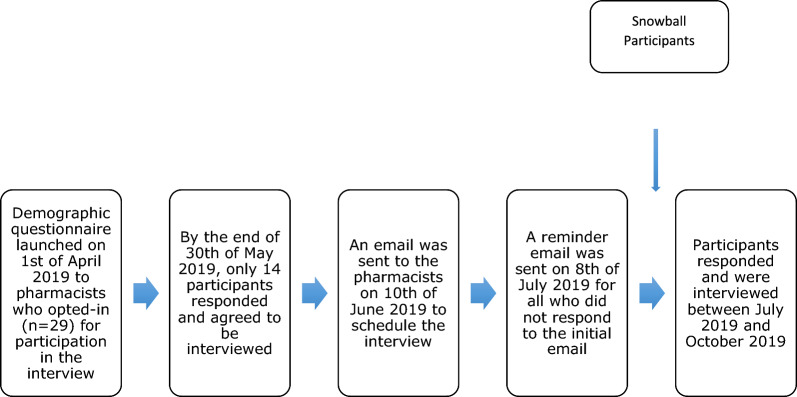
Recruitment process of the interview participants

Figure 1 shows the study recruitment process. An email invitation was sent to all 29 potential participants giving details of the interview purpose, including a brief demographic sampling questionnaire. Two reminder emails were sent at 4-weekly intervals. A subsequent email was sent to those responding (n = 14) to plan the interviews which took place between July and October 2019. Of the 14 expressing interest, 12 were interviewed, with 2 not responding after the expression of interest. Snowball sampling was used to help identify additional participants (n = 2) by asking those interviewed to recommend pharmacists matching the inclusion criteria. The intention was to achieve data saturation, by applying the approach of Francis et al., using an initial analysis sample of ten and a stopping criterion of three [32].

### Interview schedule development

The semi-structured interview schedule was based on barriers and facilitators identified from a related systematic review and survey of UKRPG members [3, 26]. The schedule was grounded in CFIR to focus the content on domains and constructs related to implementation [29]. Prior to use, the schedule was reviewed for credibility by research team members with expertise in qualitative research and renal clinical pharmacy. This was followed by ‘Think Aloud’ testing [33] prior to piloting with a pharmacist independent prescriber member of the UKRPG. As no modifications were made post-pilot, the data were included in the analysis. The semi-structured interview questions were mapped to the CFIR domains and constructs.

### Data generation

Interviews of between 20 and 50 min and were digitally recorded, transcribed verbatim and verified to ensure accuracy of transcription.

### Data analysis

The interview data were thematically analysed according to the steps described by Braun and Clarke [34]. Transcripts were imported to NVivo® 11 software [QSR International Pty Ltd. 2017] for data management. The steps in the analytical process included: 1. data familiarisation through re-listening of audio recordings and accuracy checking of transcriptions followed by reading and re-reading of the transcripts; 2. Inductive generation of initial codes to data excepts (quotes) using the CFIR domains and constructs as the chosen theoretical framework; 3. Determination of themes relevant to the aims and objectives and context of this study through generation of a framework matrix, consideration and team discussion of the allocated coded data excerpts; 4. Review of the themes to explore consolidation through consideration of similarities and differences within and across the themes; and 5. Consolidation of themes to define the ‘essence’ of the findings and focus on categorisation of themes to CFIR related barriers and / or facilitators with inclusion of a final cross-check for relevance to the research aims and objectives set. Each step in the analysis was independently conducted by two researchers with a third involved in cases of disagreement.

## Results

### Prescribing practice

Fourteen interviews were conducted to reach data saturation. Interviewees’ demographic details are given in Table 1; 9 were based in England, 3 in Scotland and one each in Wales and Northern Ireland. All were practising in secondary care with some also practising in primary care (n = 2) and community pharmacy (n = 3). Within secondary care, most prescribed a full range of renal medicines for inpatients and outpatients and some prescribed specific classes of medication (e.g., tolvaptan) in clinic settings.

**Table 1:** Interviewees’ demographics (N = 14)

Characteristic	Number (%)
*Sex*	
Male	3 (21)
Female	11 (79)
*Age range*	
31–40	7 (50)
41–50	5 (36)
51–60	2 (14)
*Years of experience as pharmacist*	
6–10	2 (14)
11–15	5 (36)
16–20	2 (14)
More than 20	5 (36)
*Years of experience as prescriber*	
Less than 1	1 (7)
1–5	5 (36)
6–10	0 (0)
11–15	7 (50)
16–20	1 (7)

Table 2 summarises the themes, categorised as facilitators or barriers, aligned to CFIR domains and constructs.

**Table 2:** Themes, categorised as facilitators or barriers, aligned to CFIR domains and constructs

CFIR domain	CFIR constructs		Themes	Facilitator/Barrier
Innovation characteristics	Intervention source		Prescribing by pharmacists: arisen from a number of different sources	Facilitator
	Relative advantage		Advantages of pharmacist prescribing role	Facilitator
	Design quality & packaging		Prescribing within competencies Replicating exemplar models Prescribing aligned with NHS Trust / Organisation needs	Facilitator Facilitator Facilitator
Outer setting	Patient needs & resources		Pharmacist prescribing appreciated by, and accessibility for, patients Learning and development needs	Facilitator Facilitator / Barrier
	Cosmopolitanism		Collaboration with external professional bodies Alliance with other units within the Trusts Digitalisation to centralise the service	Facilitator Facilitator Facilitator
Inner setting	Networks & communications		Wide range of communication within Trust Good network within organisation	Facilitator Facilitator
	Readiness for implementation	Available resources	Limited funding Personnel shortage Time to prescribe Training resources needed Need for physical space to practice New technologies needed	Barrier Barrier Barrier Barrier Barrier Barrier
Characteristics of individuals	Self-efficacy		Awareness of self-competencies Experience	Facilitator Facilitator/Barrier
	Individual Identification with Organisation		Supported by organisation	Facilitator
Implementation process	Planning		Pharmacist prescribing implementation planning Development of prescribing practice Stakeholders’ engagement importance	Facilitator/Barrier Facilitator Facilitator/Barrier
	Executing		Variation in prescribing models	Facilitator
	Reflecting & Evaluating		Monitoring of prescribing practice Development of patient feedback systems CPD/reflection and work-based appraisal systems in place	Facilitator/Barrier Facilitator Facilitator

### Themes aligned to CFIR domain and constructs

1. CFIR domain, Innovation characteristics (key attributes and features of interventions that influence implementation success).

*Intervention source*

*Theme, ‘Pharmacist prescribing need: arisen from a number of different sources’.*

Many highlighted that the intervention was a UK government legislative initiative to meet the demands of service providers.

*“…the need to make sure that pharmacy … part of the solution to meet the demand in the NHS by being professionally integrated into frontline services, the government extending the prescribing roles to other healthcare professionals…” Pharmacist 5*

There had been a transition from supplementary to independent prescribing as services developed.

*“Initially it was supplementary….and now because I’ve been here so long, all doctors accepted that I do prescribing independently as much as the doctors.” Pharmacist 2*

In the community setting, the intervention was also facilitated by contractual changes for general practitioner (GPs).

*“Generally, probably the changes in the GP contracts that asking to get community pharmacist prescribing…it just depends on where you are.” Pharmacist 7*

*Relative advantage*

*Theme, ‘Advantages of pharmacist prescribing role’.*

Pharmacist prescribing was considered advantageous for several reasons, with reduction in doctors’ workload a facilitator, allowing them to deal with more complex cases by sharing prescribing responsibilities.

*“Services are being redesigned and the ability to have more advanced practice, being able to take chronic disease management away from physicians…allowing more time for them to deal with complex cases.” Pharmacist 5*

*Design quality and packaging*

*Themes, ‘Prescribing aligned with organisation’s needs’.*

The implementation of prescribing practices varied across different geographical regions.. Obtaining a prescribing qualification was an important aspect to start the practice.

*“We just have a cohort of prescribers, and bits of prescribing document within the Trust and says that you can use your prescribing qualification in these circumstances.” Pharmacist 10*

2. CFIR domain, Outer setting (features of the external environment that have an influence on implementation).

*Patient needs and resources*

*Theme, ‘Pharmacist prescribing is accessible for, patients’.*

Many discussed patients’ needs and accessibility of prescribing as key drivers for implementation.

*“I’ve managed to establish a telephone tolvaptan clinic. So, we have some patients that were driving for an hour to come to clinic every month, which was a long time. So, managed to negotiate with the consultants to set up a telephone clinic” Pharmacist 3*

*Theme, ‘Learning and development needs’.*

While pharmacist learning and development needs and meeting these needs was a facilitator of implementation, the funding required to meet these ongoing needs in specific areas was also considered to be barriers by some.

*“In terms of barriers, I would say, having been able to do the clinical skills course, which wasn’t previously funded for pharmacists, that would have helped building the confidence and allowing you to have an extra skill” Pharmacist 9*

*Cosmopolitanism.*

*Themes, ‘Collaboration with external professional bodies’.*

Many discussed how external, independent organisations had positive impacts on their prescribing practice, with challenges from other organisations.

*“I get a lot of good ideas from the UKRPG and at RPS* [Royal Pharmaceutical Society] *conference…it’s always good to reach colleagues there, but always a challenge that other units are more advanced in prescribing in specialised clinics…” Pharmacist 6*

3. CFIR domain, Inner setting (features of the internal environment that have an influence on implementation).

*Network and communications.*

*Theme* ***‘*** *Wide range of communication within organisation’.*

Within the organisation there were various forms of formal and informal communication related to prescribing practice information or updates. Verbal communication between healthcare professionals were described in a positive way.

*“I think obviously because I’ve built a rapport with the team…the thing that helped me develop the practice because you can learn from each other.” Pharmacist 13*

A few interviewees emphasised circulation of written communications in the form of emails or bulletins to share prescribing related information.

*“Communication bulletins come up via email and on the website in terms of prescribing errors that we think would have an impact across the organisation so shared learning from errors.” Pharmacist 12*

*Available resources.*

*Themes ‘Limited fund’, ‘Personnel shortage’, ‘Time to prescribe’, ‘Training resources needed’.*

The main hindrance in terms of resources was the availability of funds, time, and personnel to enable the expansion of the prescribing service.

*“If you get resources, it will help you. If you don’t get resources, they are hindrance, so money, time, personnel, if they want to expand things, they have got to give you time and money for resources.” Pharmacist 1*

However, in relation to a key theme on ‘Training resources needed’ there was general agreement across the interviewees that the independent prescribing course was widely supported by the organisation which aided prescriber development. There were also other prescribing related courses that were available to the pharmacists to advance their prescribing skills.

*“There’s a course at* [a hospital name]*, where you can do an advanced clinical practitioner skill. So, I think because you see your patients, and you do a small bit on physical assessment but I wouldn’t be an expert” Pharmacist 8*

4. CFIR domain, Characteristics of individuals (key characteristics and features of individuals involved in the implementation success)

*Self-efficacy.*

*Themes ‘Awareness of self-competencies’, ‘Experience’, ‘Willingness to prescribe’.*

Interviewees were aware of their own abilities to be able to provide prescribing services, highlighting that they prescribed in their area of competence.

*“Unless it was fairly simple stuff, I don’t tend to get too involved in complicated stuff, I would leave that up to the medical staff. I’m not really too keen on prescribing for patients that I don’t really know that well” Pharmacist 1*

Some highlighted that they were aware of the need to develop additional skills to allow them to meet the specific needs of CKD patients.

*“We’ve got these pharmacological skills so we can develop interactions and things, I think, actually that's the benefit. So, having those, that clinical knowledge means that our patients who are prescribed tolvaptan are kept safe, so something doesn’t interact with it…” Pharmacist 9*

A few interviewees felt that they needed to further transfer their prescribing roles into more clinic settings and to more specialised areas of practice.

*“I think definitely will be clinic, I think what* [colleagues name] *doing there, heart failure, I think that will be the future. I think, you want specialist pharmacist doing specialist clinics.” Pharmacist 1*

*Individual identification with organisation.*

*Theme ‘Supported by organisation’.*

Interviewees acknowledged the positive level of commitment from their organisations.

*“I was fully supported by the department, everybody was very keen for me to do it, and yeah, I have not looked back, it's been great.” Pharmacist 14*

*Other personal attributes.*

*Themes ‘Consultation and social skills essential’, ‘Awareness of strengths and limitations’, ‘Willingness to learn and develop’.*

Some felt that their experience as a pharmacist advanced their practice as prescribers.

*“It very much depends on the competencies of the pharmacist, and I’m lucky that I’ve been in my area a long time and feel competent, most of the time, and if I don’t, then I would always have a discussion with medical staff.” Pharmacist 4*

5. CFIR domain, Process (key activities of implementation process)

*Planning.*

*Theme ‘Non-medical prescribing implementation planning’.*

None of the interviewees were particularly aware of the early planning processes to support pharmacist prescribing implementation. Some highlighted current organisational wide plans to further develop prescribing practice, increasing capacity by supporting the training and practice of pharmacist prescribers.

*“There has been a drive within our department, to get prescribers, probably about two years ago, we probably had five to ten prescribers, but over the last three years, there has been some different drive where they need to kind of boost prescriber numbers, so was all building upon numbers.” Pharmacist 10*

Some noted that the plan was framed according to the needs identified in each organisation.

*“It looks at the needs for the department so if we need more like help, you know, surgical admissions obviously the surgical pharmacist would take priority, and the admissions pharmacist because that’s when we have more of the clerking in issues, and then it is funnelled down in case of priority and need.” Pharmacist 13*

*Reflecting and evaluating.*

*Themes ‘Regular monitoring of prescribing practice’, ‘Internal/ external processes’, ‘Need to develop patient feedback systems’, ‘CPD/reflection and work-based appraisal systems in place’.*

While interviewees described various activities to allow reflection and assessment of their prescribing practice, they mostly relied on peer review to assess prescribing efficiencies or identify any errors.

*“We do a peer review every so often,* [a colleague] *peer reviewed me, she came through, and I showed her three Kardexes* [inpatient prescriptions]*. I did the same for her and we evaluated each other.” Pharmacist 1*

The electronic prescribing system within organisations was used to identify issues related to prescribing.

*“We have an electronic prescribing system, so we can see a log of what we have prescribed and what changes we have amended, literally I press the button it goes through the IT we can audit our work.” Pharmacist 12*

Others described the aspiration of an auditing process to assess prescribing efficiencies and sharing these data locally or nationally.

*“I guess maybe it would be more useful to have a more formal method of audit and reporting. I must say, I have, have a look at my own prescribing myself using the electronic system but it probably would be helpful if we had a formal way of making sure that everybody did that, and that we shared all the learning formally.” Pharmacist 11*

## Discussion

### Key findings

Interviewees had experience of prescribing in inpatient and outpatient settings and described a number of facilitators and barriers associated with prescribing implementation. They were largely positive regarding the development and implementation of their prescribing practice. Themes of facilitators and barriers emerged across all 5 CFIR domains. Key implementation facilitators mapped to aspects of the CFIR domain of inner setting (e.g., organisational support and communication). Key barriers were also related to inner setting domain, specifically the need for adequate structural and financial resources.

### Strengths and weaknesses

This qualitative study provided in-depth, rich data exploring implementation facilitators and barriers of very experienced pharmacist prescribers. It is the first published qualitative study to focus on pharmacist prescribing in CKD patients. A further strength is the use of implementation theories to underpin data collection, analysis and interpretation, thereby providing a more comprehensive portrayal of implementation. This allowed detailed and systematic consideration of all aspects of facilitators and barriers to implementing prescribing practice for patients with CKD and to enable meaningful contextualised analysis of the findings. Rigour was enhanced by consideration of elements of research trustworthiness throughout. These included credibility promoted through the use of the theoretical framework, expert review of the interview schedule; dependability through the use of established and well-documented research approaches; and transferability through description of the research setting and participants [35–37].

The main limitation is that the data were collected from members of a specialist group based in the UK hence the findings may lack transferability to non-group members practising in renal disease and those beyond the UK. Nonetheless, careful attention has been given to detailing the research context, methods employed, and characteristics of the interviewees, facilitating readers in assessing the probable applicability to their respective contexts and populations.

### Interpretation

A cross-sectional survey of UKPRG members highlighted the diversity of clinical activities for patients with CKD but provided limited details of pharmacist prescribing implementation [26]. The findings of this qualitative study complement the cross-sectional results, providing in-depth understanding of implementation facilitators and barriers. While it is apparent that pharmacist prescribing practice has significantly evolved from the early years of implementation of first supplementary and then independent prescribing [15, 16, 38], the major barrier relating to the inner setting, i.e., the need for adequate structural and financial resources remains [3, 26]. This has the potential to negatively impact wide scale implementation and sustainability of pharmacist prescribing. There are, however, key national UK developments which may help to alleviate this situation hence helping to diminish this barrier. Of note, UK government policies which prioritise the development of the pharmacist prescribing role [22–25] and current early year UK undergraduate students will have independent prescribing status on graduation. These initiatives may overcome the financial and human resource issues identified in this and other studies [13]. A study by Forsyth et al. discusses the integration of the four-pillar of the pharmacist workforce model that focuses on clinical practice, leadership, education, and research, which are essential for the comprehensive development of pharmacists’ roles and highlights the structural barriers that pharmacists face [39]. Although pharmacists have the skills and knowledge to overcome many barriers independently, the study emphasises that without adequate support in terms of resources and systemic changes, individual efforts may be inadequate. Addressing these issues requires a coordinated approach involving policy changes and organisational support, as pharmacists alone may not be able to overcome structural barriers due to issues beyond their control [39].

Early planning of the implementation process is essential and the use of the CFIR framework at an early stage is advocated to identify implementation facilitators to be emphasised and barriers to be addressed prior to implementation and scaling [29]. While the facilitators and barriers identified in this study relate to CKD, they may also be relevant to pharmacist prescribing implementation in other chronic conditions hence are worthy of further discussion. Facilitators were evident across all CFIR domains with recognition of the evidence base of the benefits pharmacist and more generally non-medical prescribing [40] along with the defined service models [26] on which others could base their own practice. Organisational support at local, national, and professional levels was also a key facilitator, reflecting aspects of CFIR inner and outer settings. Similarly, interviewees highlighted the perceived positive impact on implementation of intra and interprofessional communication and trust, as reported by others [41–45]. It is encouraging that prescribing was noted to be within areas of competence, with recognition of the need for CPD and continuous reflection. There was, however, an acknowledged need to develop more robust prescribing monitoring and feedback systems which could perhaps be developed at a national level. These will be of particular importance with the emergence of pharmacist prescriber status on graduation. Several earlier studies have reported early career pharmacists’ cautious approaches to considering prescribing practice [46, 47]. The need for advances in digitalisation to further support implementation are particularly interesting with reports of the integration of the services to improve access to clinical information and enhance prescribing [47, 48]. Bailey et al. highlight that progressing to pharmacist prescribing requires both individual and institutional changes, and that Scotland’s new national pharmacist career pathways and professional curricula aim to support skill development hence should be linked to career rewards for successful implementation [49].

### Further research

Future research needs to focus on approaches to overcome the barriers of implementing prescribing services for patients with CKD. There may be merit in considering service standard setting through consensus-based research with leaders in CKD pharmacist prescribing and other related disciplines. This could also be extended to include other prevalent chronic conditions.

## Conclusion

This theory-based study has provided insights into the various facilitators and barriers associated with the implementation of pharmacist prescribing within the context of CKD. Key facilitators and barriers reported may be considered in the process of improving the current practice. There is a need for a comprehensive assessment of the crucial resources essential for the successful implementation of prescribing practices during the initial phases of planning and development.

## Supplementary Information

Below is the link to the electronic supplementary material.

Supplementary file1 (DOCX 14 KB)
