# Supplementary material for: A qualitative, theory-based exploration of facilitators and barriers for implementation of pharmacist prescribing in chronic kidney disease
Source: Int J Clin Pharm. 2024 Sep 4;46(6):1482–91. doi: 10.1007/s11096-024-01794-y (PMC11576801; doi:10.1007/s11096-024-01794-y)
Supplement: Supplementary file 1 — Supplementary file1 (DOCX 14 KB) [file 11096_2024_1794_MOESM1_ESM.docx]

**Table 1: Interview questions mapped with CFIR domains.**

| **Related CFIR domains** | **Core questions** | **Probing questions** |
| --- | --- | --- |
| **Innovation characteristics** | What do you feel are the key factors that have influenced implementation of prescribing practice, generally and in relation to your own practice? | How do you feel you have used evidence to develop your practice?  How do you feel that your prescribing has changed your practice? What about the impact on patients?  Do you feel that your prescribing practice has changed or developed since you started?  What is the complexity of your prescribing practice: consider clinical complexity and logistics?  What about the costs and savings associated with providing a prescribing practice? |
|  | What do you feel works very well and what needs to improve regarding your prescribing practice? |  |
| **Characteristics of individuals** | How do you feel your personal characteristics have helped develop and implement prescribing practice for CKD? | How you feel you complement other in the multidisciplinary team in relation to your prescribing?  How confident are you with your prescribing?  Are you considering developing or changing any aspects of your prescribing practice?  Any other traits you have that suit your prescribing practice? |
|  | How you see your prescribing practice developing in future? |  |
| **Inner setting** | What are the barriers or facilitators, within your organisation, that have helped or hindered the development of prescribing practice generally and in your own practice? | What factors within your organisation do you feel have helped or hindered developments?  How communications within your organisation around the development of prescribing practice take place?  Do you receive any support for your prescribing role?  What about how nonmedical prescribing is welcomed, encouraged, supported?  What happens to cover prescribing practice when a colleague is absent? |
|  | What advice you would give to others who are considering setting up a prescribing service? Are there any pitfalls you should avoid? |  |
| **Implementation Process** | How was pharmacist prescribing planned for and implemented within your organisation? | How were you and colleagues involved with this? ‘project champions’  Was there any external influence on this?  How do you assess or evaluate your prescribing practice in term of safety, effectiveness, and cost effectiveness? |
| **Outer setting** | What about external influences on the development and implementation of pharmacist prescribing in your organisation generally and in your own prescribing practice? | Do you feel that colleagues in other organisations are ahead in implementing pharmacist prescribing in their practice?  Does any external body or organisation influence your prescribing practise?  Can you tell me about any other external factors affecting your prescribing practice? |
